# Hydrological legacy determines the type of enzyme inhibition in a peatlands chronosequence

**DOI:** 10.1038/s41598-017-10430-x

**Published:** 2017-08-30

**Authors:** Samuel Alexander Festing Bonnett, Edward Maltby, Chris Freeman

**Affiliations:** 1 Department of Applied Sciences, Faculty of Health and Applied Sciences, University of the West of England, Frenchay Campus, Coldharbour Lane, Bristol, BS16 1QY UK; 2 School of Environmental Sciences, University of Liverpool, Merseyside, L69 3BX UK; 3 School of Biological Sciences, Wolfson Carbon Capture Laboratory, Brambell Building, Bangor University, Deiniol Road, Bangor, LL57 2DG UK

## Abstract

Peatland ecosystems contain one-third of the world’s soil carbon store and many have been exposed to drought leading to a loss of carbon. Understanding biogeochemical mechanisms affecting decomposition in peatlands is essential for improving resilience of ecosystem function to predicted climate change. We investigated biogeochemical changes along a chronosequence of hydrological restoration (dry eroded gully, drain-blocked <2 years, drain blocked <7 years and wet pristine site), and examined whether hydrological legacy alters the response of β-glucosidase kinetics (i.e. type of inhibition) to short-term drying and waterlogging. In the dry eroded gully at depth, low phenolic concentrations were associated with enhanced β-glucosidase enzyme activities (*V*_*max*_) but short-term drying and waterlogging caused a significant increase of dissolved organic carbon (DOC) and phenolics associated with increases in *V*_*max*_ (enzyme production) and *K*_*m*_ (indicative of competitive inhibition). Inhibition within the drain blocked and pristine sites at depth exhibited non-competitive inhibition (decreased *V*_max_), whilst uncompetitive inhibition (decreased *V*_*max*_ and *K*_*m*_) occurred in surface peat explained by variation in humic substances and phenolics. These results suggest that loss of carbon by short-term drought or rewetting may occur from sites with a legacy of drought due to the release of non-inhibitory phenolics that permits enhanced enzyme activity.

## Introduction

Peatland ecosystems contain one-third of the world’s soil carbon store and are therefore a significant component of the global carbon cycle^1^. Historically, peatlands have been drained for forestry, agriculture and peat harvesting, which has led to degradation and potential loss of a variety of ecosystem services including carbon sequestration and potable water quality^2^. There are also concerns that at high latitudes, drought frequency and severity is predicted to increase^3^, and there is evidence that this is already increasing losses of carbon dioxide (CO_2_) and dissolved organic carbon (DOC)^4^. Indeed, drought and drainage combined with warming have resulted in degradation of more than 11% of global peatlands switching these carbon sinks to carbon sources^5^.

Peatland restoration aims to return these ecosystems to a defined pristine or pre-determined less degraded state. Peat quality depends on physical, chemical, and biological properties, especially the activities of hydrolytic enzymes that are frequently measured to evaluate microbial and ecosystem function^6, 7^. Key processes in restoration include identifying and understanding the biogeochemical mechanisms that enhance and inhibit the decomposition of organic matter (OM) in ombrotrophic peatlands so that management strategies can be developed to promote the resilience of peatland ecosystem functions to predicted climate change^8, 9^. Recent contrasting results on the effect of drought and even rewetting on decomposition in peat particularly dissolved organic carbon (DOC) release, raise uncertainty on the conventional theory that anoxia is the key to carbon storage in peat^10–13^.

Long-term environmental conditions such as temperature^14, 15^, vegetation^10^, acidity^16^ and particularly hydrology^17–19^ affect decomposition as well as the degree of peat formation (humification) via effects on microbial and enzymic degradation of OM^20^. The variation of the water table depth is considered as the principle driving factor influencing peatland ecosystems, compared to other environmental conditions^21, 22^. Decomposition of OM and ultimate CO_2_ release depend on the combined response of extracellular and intracellular (microbial), enzymatically mediated reactions^14^. Extracellular enzymes such as β-glucosidase catalyze the initial enzymatic hydrolysis of a variety of complex polysaccharides in peat to simple monomers (i.e. glucose) that can be transported actively and passively into microbial cells and catabolized by intracellular enzymes producing CO_2_. Extracellular enzyme catalytic activities therefore limit the rate of decomposition^17, 23^ and are markedly affected by site-specific factors such as moisture, nutrient availability, inhibitors and other site parameters^24, 25^. Freeman *et al*. and Fenner & Freeman describe in detail the enzyme latch mechanism responsible for decomposition of peat, in which oxygen (O_2_) constraints on the enzyme phenol oxidase prevent the decomposition of peatland carbon due to phenolic compounds inhibiting hydrolase enzymes^17, 18^. This enzymic latch sits within a regulatory pathway of process-specific limitations, which are sequentially removed as drought proceeds, constituting a biogeochemical cascade with potent positive feedbacks to carbon loss^18^. Following drought, the cascade consists of (1) ingress of O_2_ enabling increased microbial growth rates that results in (2) increased synthesis of phenol oxidase. This increase in activity causes (3) a decline in inhibitory phenolics that (4) stimulates microbial growth and metabolism and edaphic hydrolases. Increased activity (5) stimulates release of carbon and nutrients following re-wetting of peat, further enhancing (6) microbial activity and abundance, that (7) positively feeds-back on the synthesis of hydrolases, phenol oxidases, CO_2_ emissions and also (8) edaphic enzyme activities^18^. Therefore severe drought and counterintuitively rewetting can destabilize peatland carbon stocks raising concerns for peatland restoration management under predicted climate change.

Extracellular enzymes are usually adsorbed on the surface of organic material in soil, and the heterogeneous nature of soil affects the kinetic diversity of immobilized enzymes^26^. In Michaelis–Menten kinetics, *V*_max_ defines the maximum rate of enzyme activity under saturating substrate concentrations, and *K*_*m*_ (the Michaelis constant) defines the substrate concentration at which 50% *V*_*max*_ is achieved and represents the substrate affinity of the enzyme under non-saturating substrate concentrations^27^. Soil moisture content will affect the movement of enzymes and their substrate concentrations and the diffusional limitation of the substrates may directly affect soil enzyme kinetics^28^. Changes to enzyme kinetics may reflect differences in the types of inhibition by OM and/or DOC such as competitive inhibition (where substrate and inhibitor molecules compete for the active site resulting in an increase in *K*_*m*_), uncompetitive inhibition (where an inhibitor binds to the ES complex resulting in both decreased *V*_*max*_ and *K*_*m*_) or non-competitive inhibition (where the inhibitor binds equally well to the enzyme whether or not it has already bound the substrate and decreases *V*_*max*_). Humic substances are the dominant fraction in DOC^11^ and humic acids and phenolic compounds are known to play an important role in the stabilisation or inhibition of extracellular enzymes in soils^29–31^ through complexation or covalent binding reactions that impede substrate access to the enzyme active site^32–34^. Hydrology affects humification and the self-assembly of humic substances over time (fulvic acids first, humic acids later) as redox reactions occurring under drought (oxidation) and re-wetting (reduction) events affect the formation and accumulation of humic matter (see Tan for models of humic matter genesis)^35^. Change in *V*_*max*_ and *K*_*m*_ may also reflect shifts in the microbial community structure^36, 37^ such as the bacterial-to-fungal ratio that may alter carbon allocation to the extracellular enzyme pool affecting the kinetic characteristics such as more rapid conformational changes^14^.

Understanding the effects of climate on decomposition is complicated by the relationship between direct effects, which dictate the rate responses of enzyme-catalyzed microbial processes, and indirect effects, which alter the structure and composition of microbial communities^38, 39^. The phylogenetic and functional response of microbial communities under drought are an emergent property of the ecosystem determined by life history strategies and traits^40, 41^. Hydrological impacts that modify microbial community structure can also alter intracellular allocation pathways within organisms and populations^40^. Constraints on substrate diffusion can promote the allocation of carbon to extracellular enzyme production, stimulating the decomposition of polymeric compounds to monomers for intracellular cellular metabolism^42^. Therefore, historical environmental conditions select for microbial communities that alter the production of extracellular enzymes and the decomposition of carbon^19^. “Legacy effects” represent the persistent influence of previous ecosystem conditions that reflect the history of a site^43, 44^. We hypothesize that hydrological legacy of a site (i.e. previous long-term historical exposure to drought) will alter the response of microbial decomposition and Michealis-Menten enzyme kinetics (*V*_*max*_ and *K*_*m*_) to contemporary (short-term) variation in hydrology potentially affecting the release of DOC. The impact of rewetting by ditch-blocking on DOC export in peat bogs appears dependent on the length of time following rewetting but the mechanisms responsible are not fully understood^11–13^. Recent studies show the prominent role of indirect and direct temperature effects on microbial community composition in governing decomposition rates via enzyme expression and intracellular metabolism of the products formed^15, 19^. As hydrology is arguably of more significance in peatland systems, the formation of distinct humic or phenolic compounds due to differences in hydrological legacy may play an important role in how peatland decomposition processes respond to climate change and restoration management.

**Figure 1:**
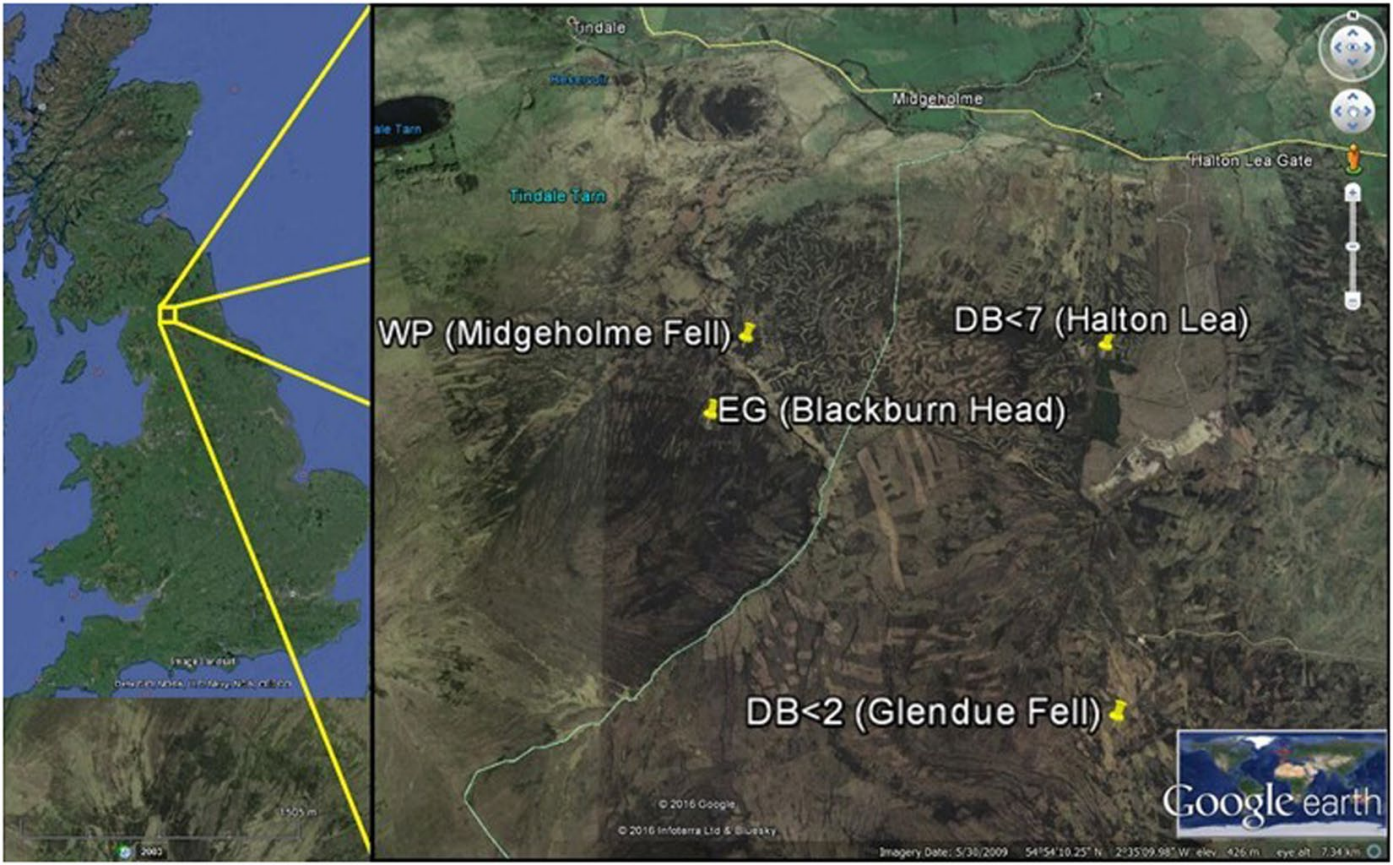
Sites located within the Geltsdale National Nature Reserve, UK that differed in long-term legacy of hydrological condition and management. Wet pristine [WP] and dry eroded gully [EG] or managed peatland (ditch-blocked <2 years [DB2] and ditch-blocked <7 years [DB7]. All images from Google Earth (© 2016 Google and © 2016 Infoterra Ltd & Bluesky).

The aim of this study was to investigate relationships between physical (hydrology), chemical (organic matter) and biological (enzymic) properties of peat across a chronosequence of peatland sites within the Geltsdale National Nature Reserve, UK that differed in long-term legacy of hydrological condition and/or restoration management. The four sites (Fig. 1) were either unmanaged (wet pristine [WP] and dry eroded gully [EG]), or managed peatland (ditch-blocked <2 years [DB2] and ditch-blocked <7 years [DB7]), located in relatively close proximity to minimise variation in local temperature and rainfall. These locations represented a potential long-term hydrological legacy in two spatial dimensions along a gradient in the order EG < DB2 < DB7 < WP with the third-dimension represented by surface (fluctuating water table) and deep (permanent waterlogged) peat. The objectives of our study were (1) to observe whether changes in the physical (hydrology), chemical (OM and DOC) and biological (microbial and extracellular enzyme) activity along the hydrological chronosequence were consistent with restoration, and (2) to examine whether hydrological restoration (“legacy”) alters the response of β-glucosidase kinetics (i.e. type of inhibition) to short-term variation in hydrology by laboratory drying or waterlogging.

## Results

### Baseline hydrological legacy effects

Table 1 shows baseline physical and chemical conditions at each site in spring 2010. In surface peat, baseline gravimetric water content was significantly lower in EG compared to DB7 (*P* < 0.022) and WP (*P* < 0.019) sites. Water content was also significantly higher at depth than the surface in the EG site (*P* < 0.007). Correspondingly, surface SOM content was significantly lower in EG (*P* < 0.005) and DB2 (*P* < 0.006) sites compared to the WP site. SOM was also significantly lower at the surface in the EG (*P* < 0.001), DB2 (*P* < 0.001) and DB7 (*P* < 0.009) sites compared to deep peat. In surface peat, the E_4_/E_6_ ratio was significantly lower within the DB7 (*P* < 0.001) site compared to the other sites that may be explained by significantly higher pH (*P* < 0.012). However, the E_4_/E_6_ ratio was significantly lower at depth compared to the surface in the EG (*P* < 0.001), DB2 (*P* < 0.002) and WP (*P* < 0.001) sites. Additionally, humification degree (Abs 540 nm) was significantly higher at depth compared to the surface in the EG (*P* < 0.017), DB7 (*P* < 0.022) and WP (*P* < 0.003) sites.

**Table 1:** Baseline climatic and physical characteristics.

	Eroded gully (EG)	Ditch-blocked<2 years (DB2)	Ditch-blocked<7 years (DB7)	Wet pristine (WP)
Location name	Blackburn Head	Glendue Fell	Haltonlea Fell	Midgeholme Fell
Sampling date	12/05/10	14/04/10	21/04/10	18/05/10
Coordinates	N 54 54.429 W 002 35.261	N 54 52.371 W 002 36.231	N 54 54.265 W 002 32.513	N 54 54.566 W 002 34.404
Air temperature (°C)	7.8 (1.09)	8.9 (0.79)	11 (1.20)	13 (1.30)
Peat temperature (°C)				
- Surface	5.4 (0.14)	4.6 (0.06)	5.2 (0.07)	6.6 (0.08)
- Deep	5.8 (0.03)	4.7 (0.05)	5.3 (0.02)	5.9 (0.06)
Gravimetric water content (g H_2_O g^−1^ peat)				
- Surface	7.38 (0.741)^1a^	7.84 (0.212)	10.48 (0.758)^2^	10.54 (0.409)^2^
- Deep	10.92 (0.280)^b^	7.88 (0.632)	9.87 (0.828)	8.72 (0.584)
pH				
- Surface	3.8 (0.02)	3.9 (0.04)	4.0 (0.03)	3.7 (0.08)
- Deep	3.8 (0.02)	3.9 (0.04)	4.0 (0.03)	3.9 (0.01)
Bulk density (g cm^3^)				
- Surface	0.11 (0.013)	0.11 (0.004)	0.08 (0.004)	0.08 (0.004)
- Deep	0.09 (0.003)	0.12 (0.014)	0.09 (0.007)	0.10 (0.010)
SOM (% LOI)				
- Surface	96.3 (0.260)^1a^	96.4 (0.368)^1a^	97.4 (0.244)^a^	97.8 (0.250)^2^
- Deep	98.7 (0.046)^b^	98.2 (0.127)^b^	98.5 (0.183)^b^	98.6 (0.127)
E_4_/E_6_				
- Surface	6.16 (0.250)^1a^	6.52 (0.522)^1a^	4.21 (0.348)^2^	6.49 (0.263)^1a^
- Deep	3.78 (0.122)^b^	4.72 (0.108)^b^	3.84 (0.172)	4.42 (0.078)^b^
Humification (Abs 540 nm)				
- Surface	0.18 (0.030)^a^	0.19 (0.040)	0.16 (0.018)^a^	0.14 (0.016)^a^
- Deep	0.31 (0.021)^b^	0.22 (0.022)	0.29 (0.012)^b^	0.29 (0.019)^b^

Significant differences between sites (*P* < 0.05) are indicated by different numbers; depths by letters. Mean (standard error) (*n* = 4). LOI = Loss On Ignition.

**Figure 2:**
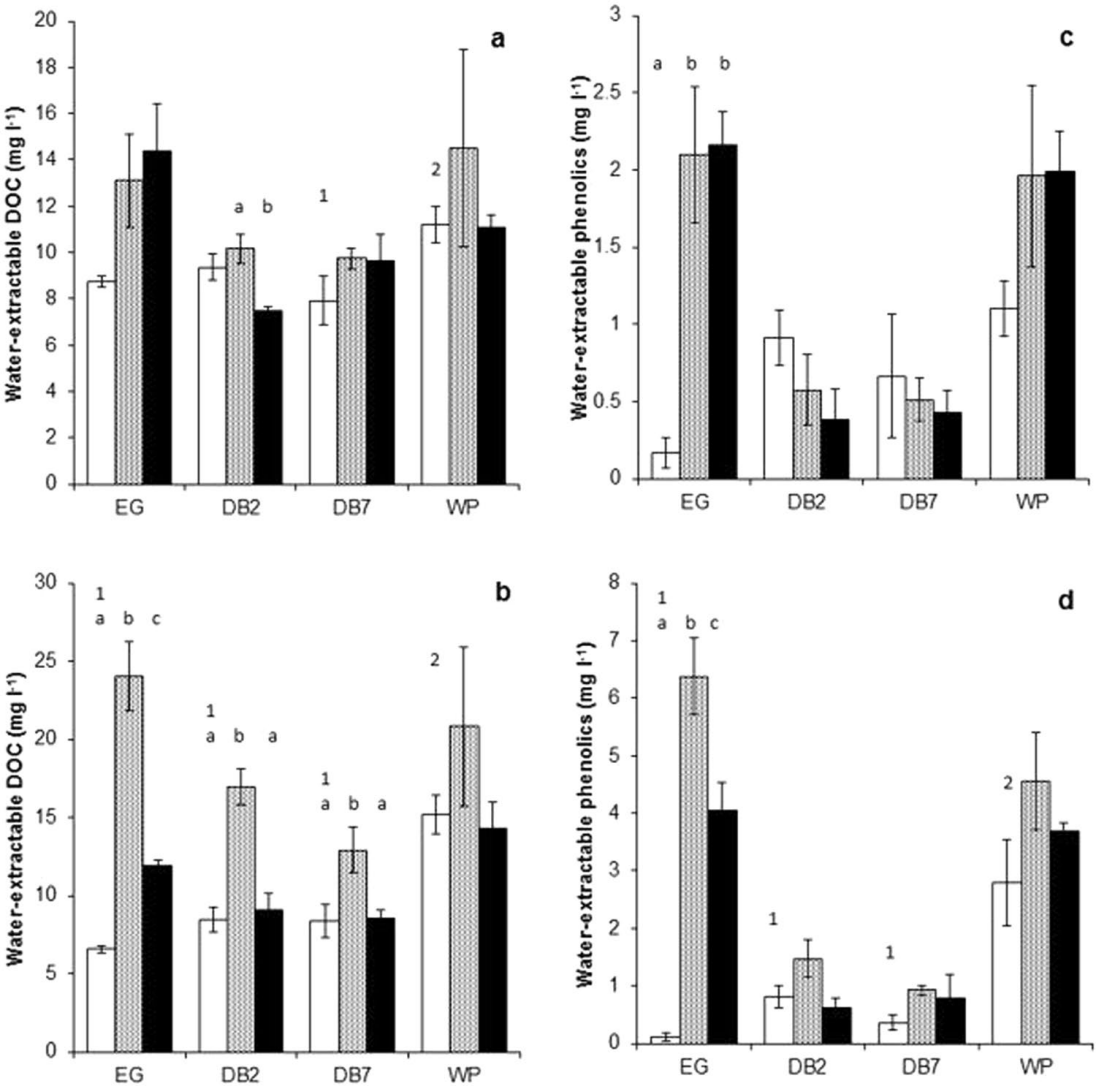
Water-extractable DOC concentration within (**a**) surface peat and (**b**) peat at depth and water-extractable phenolic concentration within (**c**) surface peat and (**d**) peat at depth. White bars = baseline measurements, grey bars = air dried treatment and black bars = waterlogged treatment. Significant differences between site baseline measurements (*P* < 0.05) are indicated by different numbers. Significant differences between baseline, dry and waterlogged conditions within each site are indicated by different letters. Mean ± standard error (*n* = 4).

There were no significant differences in baseline DOC or biological measurements in surface peat. However, at depth, baseline DOC (Fig. 2b; *P* < 0.001) and phenolics (Fig. 2d; *P* < 0.018) were significantly higher in the WP site relative to all other sites. Within the WP site, DOC was also higher at depth compared to the surface (*P* < 0.045). The phenolic/DOC ratio (Supplementary Table S1) was also significantly higher at depth in the WP site relative to the EG site (*P* < 0.002) whilst SUVA_254_ (Table S1; *P* < 0.001) was significantly higher in the WP site in both surface and deep peat. Interestingly, baseline DOC, phenolics and SUVA_254_ were significantly lower at depth in the EG site (Site*Depth interaction *P* < 0.01), but significantly higher at depth in the WP site (Site*Depth interaction *P* < 0.05).

**Figure 3:**
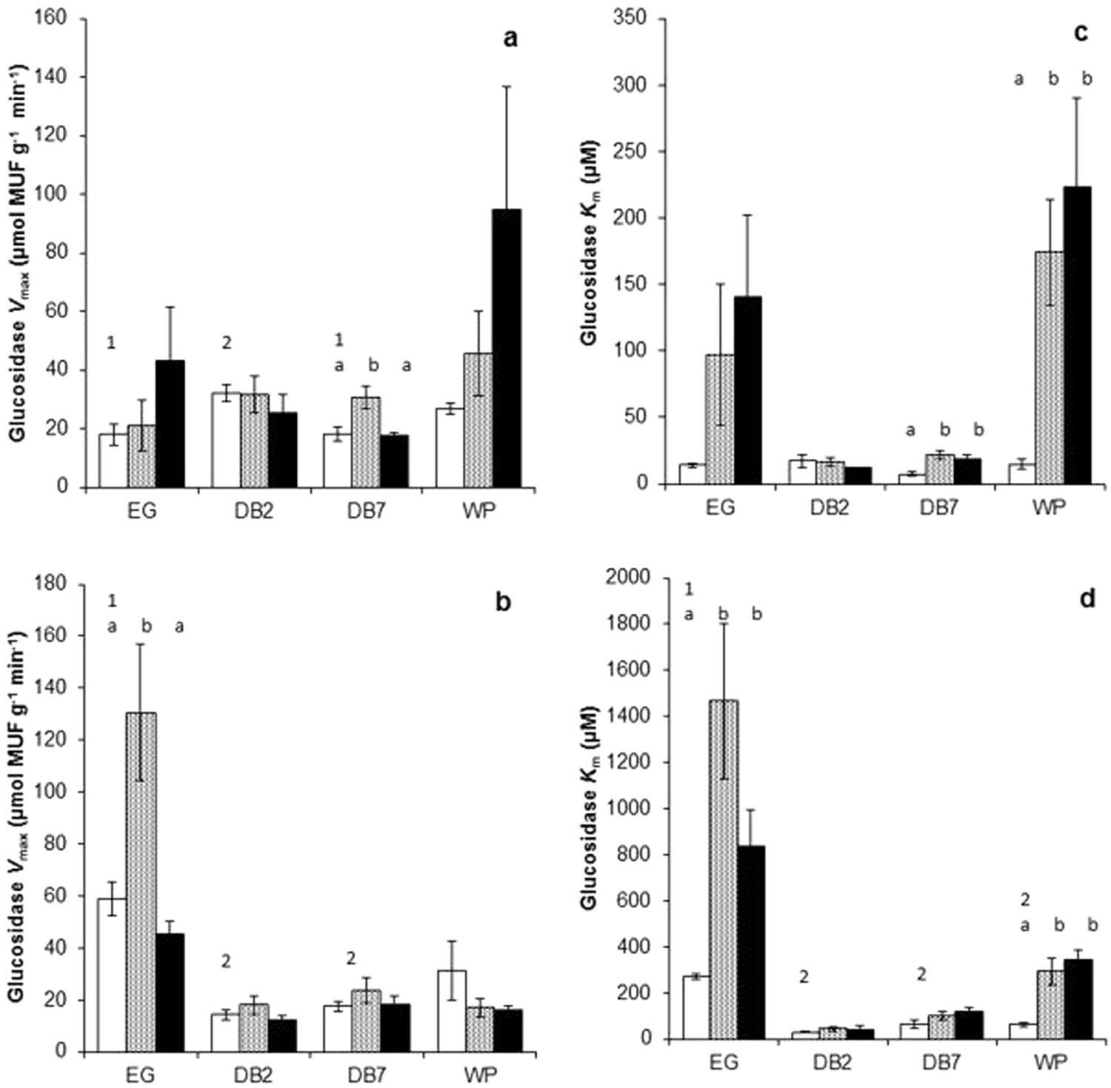
β-glucosidase activity (*V*_*max*_) within (**a**) surface peat and (**b**) peat at depth and Michaelis constant (*K*_*m*_) within (**c**) surface peat and (**d**) peat at depth. White bars = baseline measurements, grey bars = air dried treatment and black bars = waterlogged treatment. Significant differences between site baseline measurements (*P* < 0.05) are indicated by different numbers. Significant differences between baseline, dry and waterlogged conditions within each site are indicated by different letters. Mean ± standard error (*n* = 4).

Baseline heterotrophic respiration was significantly higher in the eroded gully (EG) site relative to the drain blocked <2 years (DB2) site (Supplementary Figure S1; *P* < 0.035) whilst net CH_4_ production was significantly higher at depth overall compared to surface peat (*P* < 0.05). However, baseline β-glucosidase *V*_*max*_ (*P* < 0.014) and *K*_*m*_ (*P* < 0.001) were also significantly higher in the EG site relative to all other sites (Fig. 3b,d). *V*_*max*_ was higher at depth compared to the surface in the EG site only (*P* < 0.001) whilst *K*_*m*_ was significantly higher at depth compared to the surface in the EG (*P* < 0.001), DB7 (*P* < 0.004) and WP (*P* < 0.020) sites.

### Laboratory hydrological manipulations

Air drying in the laboratory significantly reduced the water content relative to baseline and waterlogged conditions in all sites at surface (*P* < 0.001) and depth (*P* < 0.05) whilst waterlogging was not significantly different to baseline water content (Table S1). The E_4_/E_6_ ratio was increased by drying and waterlogging in the DB7 site in the surface (*P* < 0.001) and at depth (*P* < 0.006) due to the low baseline E_4_/E_6_ ratio attributed to the high pH.

In surface peat, although air dried heterotrophic respiration appears lower compared to baseline levels (Supplementary Figure S1), results were not consistently significant across sites with only the DB2 site showing a significant effect between air dried and waterlogged (*P* < 0.001) and in the WP site between baseline and air dried (*P* < 0.005). In surface peat from the EG site, air drying and waterlogging significantly increased SUVA_254_ (*P* < 0.001), phenolics (*P* < 0.001; Fig. 2c) and the phenolic/DOC ratio (*P* < 0.007). Air drying (*P* < 0.053) and waterlogging (*P* < 0.001) also increased *K*_*m*_ significantly (Fig. 3c) in the WP site that corresponded with an increase in humification (Table S1; *P* < 0.003).

At depth, heterotrophic respiration (*P* < 0.01) and net CH_4_ (*P* < 0.05) production were consistently and significantly reduced by air drying relative to baseline conditions in all sites (Supplementary results Figure S1). However, air drying significantly increased DOC (Fig. 2b), phenolics (Fig. 2d), SUVA_254_, and the phenolic/DOC ratio in the EG site only (*P* < 0.001) corresponding with a significant decrease in pH (*P* < 0.001), and an increase in glucosidase *V*_*max*_ (*P* < 0.001; Fig. 3b) and *K*_*m*_ (*P* < 0.001; Fig. 3d). Glucosidase *K*_*m*_, phenolics, SUVA_254_, and the phenolic/DOC ratio were also increased by waterlogging in the EG site (*P* < 0.001). There were no significant differences in phenol oxidase although results show potential for increase in short-drying and waterlogging within the EG site (*P* < 0.064; Supplementary results Figure S2).

### *In vitro* inhibition of pure β-glucosidase enzymes with peat extracts

**Figure 4:**
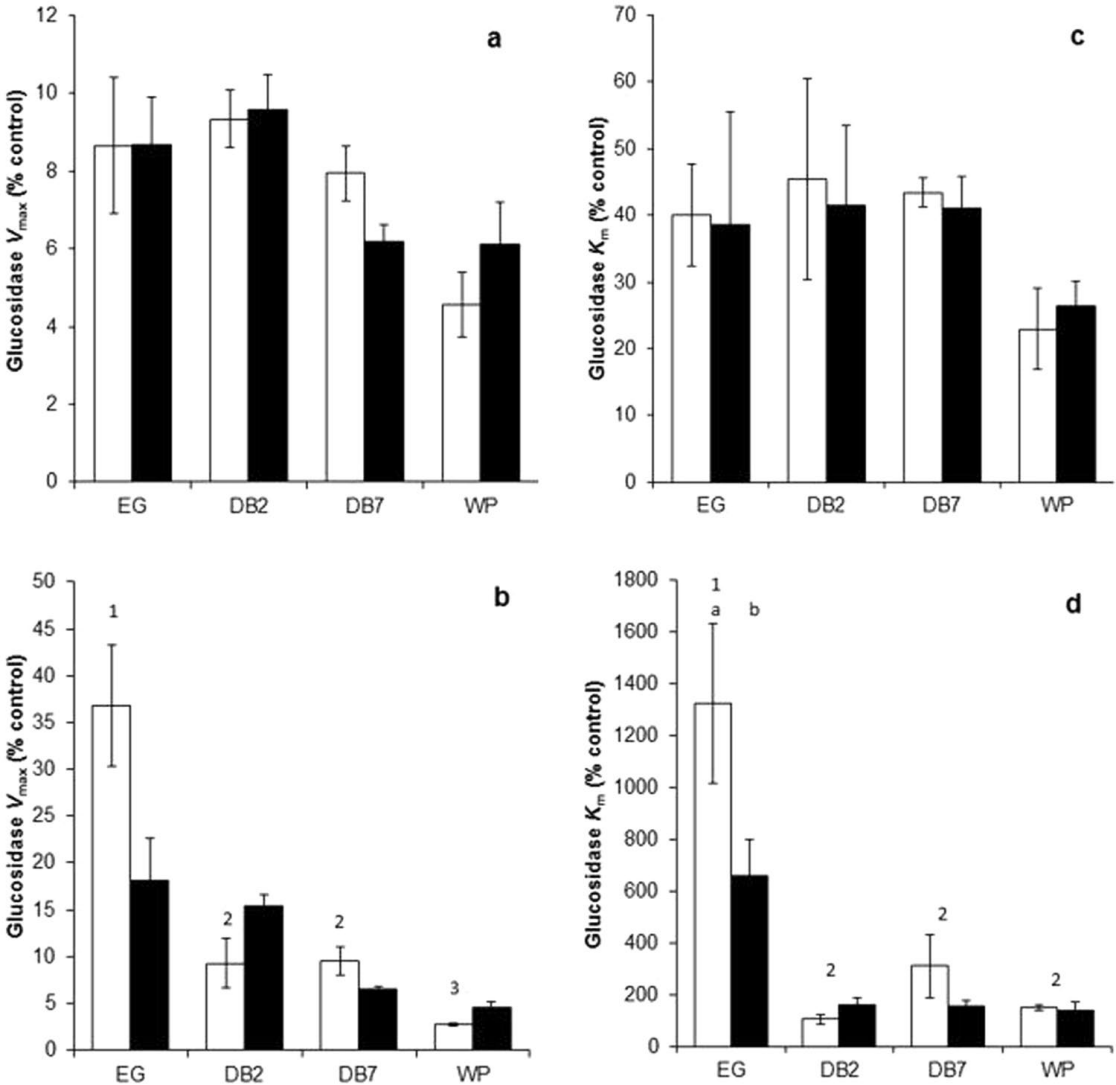
Effect of peat extract on pure β-glucosidase activity from (**a**) surface peat and (**b**) deep peat; pure β-glucosidase *K*_*m*_ from (**c**) surface peat and (**d**) deep peat. White bars = air dried treatment and black bars = waterlogged treatment. Significant differences between site baseline measurements (*P* < 0.05) are indicated by different numbers. Significant differences between baseline, dry and waterlogged conditions within each site are indicated by different letters. Mean ± standard error (*n* = 4).

To evaluate whether the degree and type of β-glucosidase inhibition (*V*_max_ and *K*_m_) differed between each site, peat extracts were incubated with 1U of pure β-glucosidase enzyme relative to a deionised water control (see Materials and Methods). Surface peat inhibited pure β-glucosidase activity (*V*_*max*_) to between 4 and 10% of the control across all sites (Fig. 4a) and *K*_*m*_ was reduced to between 22 and 46% of the control (Fig. 4b) although there were no significant differences between sites corresponding with baseline observations. Non-linear regression modelling (Table S2) and plots of *K*_*iNR*_ against [*S*] (Fig. S1) confirmed uncompetitive inhibition of β-glucosidase in surface peat. Deep peat inhibited pure β-glucosidase *V*_*max*_ to between 2 and 36% of the control across all sites (Fig. 4c). Inhibition was significantly higher in WP peat relative to the DB sites (*P* < 0.012) that were together all significantly lower than inhibition in the EG site (*P* < 0.007). There was no significant effect of hydrological treatment within sites although there was a significant interaction between site and hydrological treatment (*P* < 0.001). *K*_*m*_ was increased by deep peat from all sites although EG deep peat increased *K*_*m*_ significantly between 4 to 12 times (1326%) the other sites in the dry treatment (*P* < 0.001; Fig. 4d). There was a significant interactive effect of site x hydrological treatment (*P* < 0.049) due to significantly higher *K*_*m*_ in the air dried compared to the waterlogged peat from the EG site (*P* < 0.028). Non-linear regression modelling (Table S2) and plots of *K*_*iNR*_ against [*S*] (Fig. S1) confirmed competitive inhibition in the EG site but non-competitive inhibition in the other deep peats. Potential and *in vitro V*_*max*_ were positively correlated (*P* < 0.001; *r* = 0.764) as were potential and *in vitro K*_*m*_ (*P* < 0.001; *r* = 0.677) across all sites.

### Multivariate analyses

Multiple stepwise regression analyses were used to determine the relationship between either potential or *in vitro* β-glucosidase kinetics (*V*_*max*_ and *K*_*m*_) with physical, chemical and biological parameters using all, surface or deep datasets. As can be seen in Table 2, only *in vitro K*_*m*_ variation in surface peat could not be modelled significantly using the measured parameters, and in addition, all modelled kinetics in surface peat had low adjusted *R*_2_ values suggesting limited explained variation (approximately 22–36%). Models using only deep peat replicates explained more variation than models using data from both depths. In particular, the H^+^ ion and phenolic concentration had a positive effect on potential *V*_*max*_ and *K*_*m*_ at depth together explaining greater than 69% of the variation. *In vitro V*_*max*_ and *K*_*m*_ at depth were also related to the H^+^ ion concentration but this only explained between 33 and 46% of the variation respectively. Comparing the effect of other factors between each dataset suggests that the OM fractions may mediate effects on β-glucosidase kinetics via the type of inhibition.

**Table 2:** Multiple stepwise regression analysis for independent factor effects on β-glucosidase *V*_*max*_ and *K*_*m*_ in both potential and *in vitro* (pure enzyme) extracts using either all, surface or deep peat datasets.

Dataset	Kinetic parameter	H^+^	H_2_O	SOM	Phenolics	E_4_/E_6_	DOC	SUVA_254_	540	CO_2_	*Adi R*_2_	*P* value	*F*
All	*V*_*max*_				11.238 (0.520)	9.225 (0.268)			91.416 (0.342)		0.293	0.001	9.684
	*K*_*m*_	2323217 (0.316)			192.74 (0.864)		−24.541 (−0.338)				0.660	0.001	41.748
	*In vitro V*_*max*_				0.116 (0.889)			−0.085 (−0.537)			0.316	0.001	15.559
	*In vitro K*_*m*_	2237202 (0.462)	16.85 (0.223)		34.290 (0.234)	−64.44 (−0.275)					0.532	0.001	18.915
Surface	*V*_*max*_								122.79 (0.493)		0.217	0.004	9.615
	*K*_*m*_							38.20 (0.419)	281.64 (0.415)		0.355	0.001	9.521
	*In vitro V*_*max*_			−0.028 (−0.566)							0.297	0.001	14.107
	*In vitro K*_*m*_											n.s.	
Deep	*V*_*max*_	410835 (0.651)			9.088 (0.479)					1.150 (0.291)	0.707	0.001	25.954
	*K*_*m*_	3311465 (0.411)	41.76 (0.269)		143.85 (0.594)						0.687	0.001	23.696
	*In vitro V*_*max*_	+1815 (0.374)				−0.186 (−0.386)					0.331	0.001	8.683
	*In vitro K*_*m*_	3992021 (0.767)	37.08 (0.370)								0.456	0.001	13.986

Unstandardized coefficients and standardized β coefficients in brackets for each independent factor.

**Figure 5:**
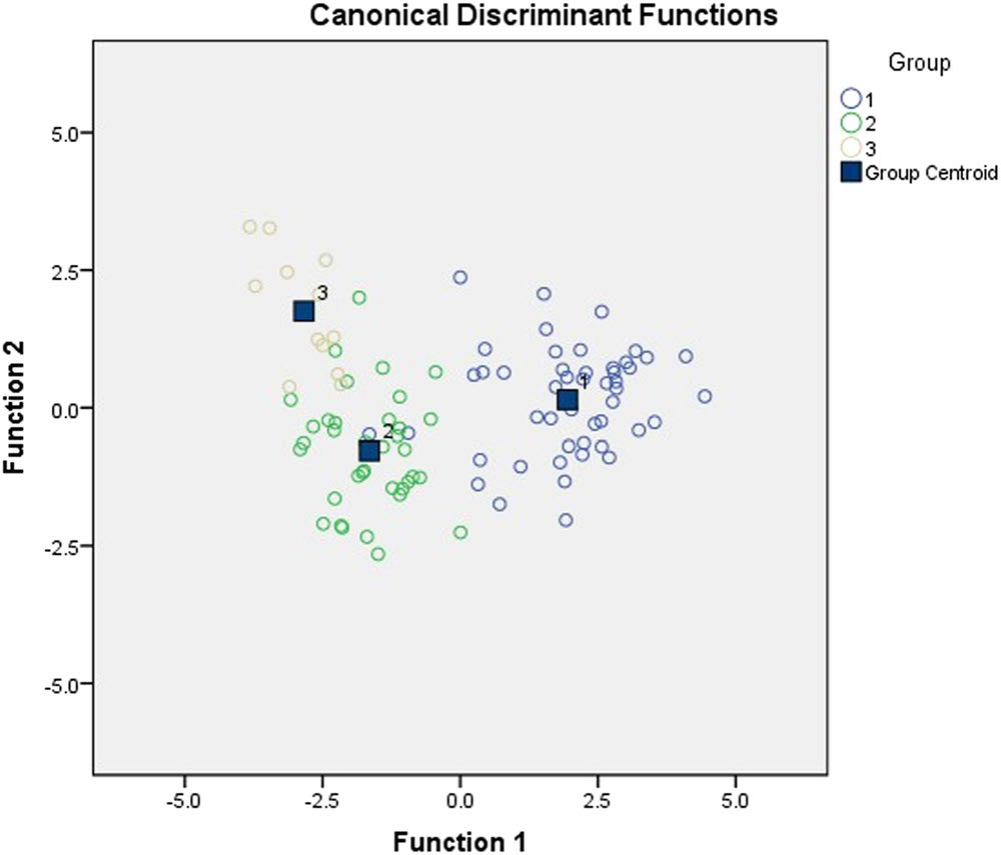
Canonical Discriminant Functions for groups according to model of enzyme inhibition. Group 1: Uncompetitive inhibition = 96.9%; Group 2: Non-competitive inhibition = 87.5%; Group 3: Competitive inhibition = 100%. Of the original grouped cases, 93.8% were correctly classified.

**Figure 6:**
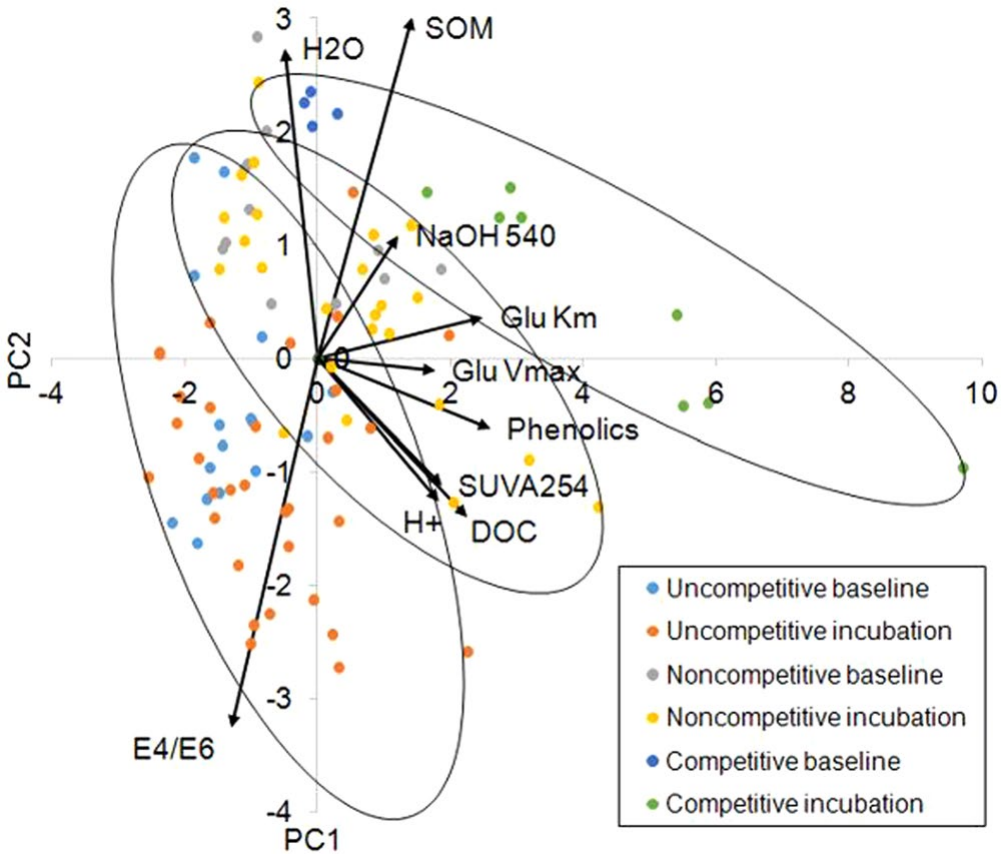
Principal Components Analysis showing enzymic inhibition (uncompetitive, non-competitive and competitive groups in circles) related to SOM and humification that is perpendicular to within group variation (baseline to hydrological incubation) related to DOC, phenolic, H^+^ ion and enzyme kinetics.

Discriminant Function Analysis revealed that the three types of enzyme inhibition (uncompetitive in surface peat, competitive at depth in the EG site and non-competitive at depth in the DB and WP sites) were best explained first by SOM (−0.731), second by the E_4_/E_6_ ratio (0.699) and third by dissolved phenolic concentration (0.307) according to the standardized canonical discriminant function coefficients in brackets (Fig. 5; Table S3) with 92.7% of original grouped cases correctly classified (Uncompetitive – 95.8%; Competitive – 75%; Non-competitive – 94.4%). Wilk’s lambdae were significant for both functions (*P* < 0.001) with function 1 explaining most of the variance. However, Box’s M test was significant (*P* < 0.001) suggesting that the assumption of homogeneity of covariance matrices was not met. Comparison of log determinants showed that the competitive inhibition group (−35.4) differed to the uncompetitive (−21.47) and non-competitive (−22.4) groups suggesting uncertainty in the attribution of factors explaining the competitive group. Figure 6 shows how the three enzyme inhibition groups are separated by two principle components that clearly relate to the variation in SOM and the E_4_/E_6_ ratio. It can be seen that DOC and the other organic fractions appear perpendicular to the enzyme inhibition functional groups that relates to the effect of laboratory hydrological manipulation, particularly in the competitive inhibition group that exhibit significant increase in phenolic compounds.

## Discussion

Our study shows that hydrological legacy alters how the functional microbial community responds to contemporary hydrological change. Physical, chemical and biological parameters varied across the chronosequence consistent with recovery from drought. The baseline results showed that decomposition of OM within surface peat had been enhanced over the long-term in the ‘drier’ EG site compared to the ‘wetter’ WP sites with the DB sites intermediate. However, in deep peat, elevated DOC, phenolics and other aromatic fractions resembled the enzymic latch mechanism with phenolic inhibition of β-glucosidase in the WP site relative to the EG site^17, 18^. Other studies have shown increasing activities of enzymes with restoration suggesting enhanced oxidative microbial capacity^6^. However, the decreasing trend in extracellular β-glucosidase at depth shown here suggests that restoration has relatively rapid effects (i.e. the DB < 2 years) on carbon decomposition. As β-glucosidase activity was low in the DB sites despite low concentrations of phenolics, we did not observe a transient gradient of change in the enzyme latch mechanism across the chronosequence and therefore step changes may have occurred following rewetting related to change in the form or molecular weight of phenolics produced (i.e. inhibitory vs non-inhibitory)^18, 45^. Indeed, these results and those discussed below regarding short-term hydrological responses, suggest that restored sites show distinct characteristics to degraded and pristine systems possibly reflecting legacy effects on the microbial community structure and function that requires further investigation.

The significant differences in decomposition pools and processes at depth was counter-intuitive as we expected sites to be similar at depth due to the constant water table (i.e. the catotelm) with hydrological legacies primarily causing differences between sites in surface peat where the water table fluctuates (i.e. the acrotelm). It is possible that the depth of the acrotelm-catotelm border differed between each site and this should be considered in future field investigations. However, deep peat is generally characterized by anaerobic conditions and older more humified carbon with slower exchange of energy and matter^46^. Indeed, Moore *et al*. found that fluvial DOC loss in tropical peatlands disturbed by drainage, deforestation and fire was primarily old (centuries to millennia) carbon from deep within the peat profile^47^. Under anoxic conditions, fermentative metabolisms are dominant, promoting the production of DOC^48^, as seen in the WP site and at depth, even though decomposition might be slowed^13^. Schiff *et al*. suggested that, above the maximum depth of the water table, the water flow is rapid enough to flush out DOC produced in surface layers but at depth, as the groundwater flow is much slower, DOC can still accumulate^49^. As DOC was lower at depth in the EG site, this suggests substantial drainage and DOC loss in the EG site over time. Kalbitz *et al*. also found greater peat decomposition in degraded peatlands resulting in lower DOC concentrations in soil solution with a higher proportion of aromatic compounds^50^. Also, Braggaza *et al*. measured enzyme activities and DOC in ombrotrophic peatlands along an altitudinal gradient of decreasing temperature and increasing water content and found enhanced β-glucosidase activity at the relatively dry low altitude peatland and phenol oxidase inhibition at the wet high altitude peatland with a decreasing gradient of DOC with altitude^51^.

The second objective was to examine whether hydrological legacy alters the response of β-glucosidase kinetics and the type of inhibition to short-term variation in hydrology. Importantly, the CO_2_ and CH_4_ results show that the drying treatments caused osmotic disruption of microbial biomass and likely lysis of cellular components particularly at depth that may have contributed to the DOC pool. Therefore these effects reflect extreme drought and therefore do not necessarily reflect moderate drought effects likely under field or predicted climate change conditions. However, the response of enzyme kinetics and DOC to extreme drought were clearly dependent on hydrological legacy as shown by the increase in *V*_max_ and *K*_m_ at the EG site associated with a positive increase in DOC and phenolics. Bouskill *et al*. found a significant increase in compounds related to metabolic and carbohydrate biosynthesis in a tropical forest soil drought experiment that suggested a metabolic response in the microbial community caused by elevated enzyme production rather than stabilization of sorbed enzymes^19^. The increase in β-glucosidase activity (*V*_*max*_) within the EG site, reflects a stimulation of microbial *de novo* synthesis that is as expected via the enzyme latch mechanism despite the drop in heterotrophic respiration^17, 18^. Newly released enzymes will be considerably more active, despite the release of phenolic inhibitors^18^. The change in β-glucosidase kinetics might also reflect the decrease in pH and increased substrate availability (affecting *V*_*max*_). Interestingly, whilst the dry EG site exhibited an increase in potential *V*_max_ and *K*_m_, our *in vitro* study showed evidence of competitive inhibition (increased *K*_m_ only) in which the inhibitor binds to the active site, reducing the affinity of the enzyme for substrate. A number of studies have linked SOM and humic complexes containing phenols to competitive inhibition of β-glucosidase^52–55^. We suggest that competitive inhibition within the ‘open latch’ EG peat may relate to humic (phenolic) substances (i.e. increase in *K*_m_) but also edaphic enzymes (i.e. increase in *V*_max_) that are less sensitive to phenolics due to stimulation of microbial enzyme expression^18^ and/or a hydrological legacy effect of less inhibitive phenolic material (<1000 AMW) due to enhanced phenol oxidase activity over the longer term^31, 56, 57^. Although no significant change in phenol oxidase activity was observed, there was evidence of elevated phenol oxidase under extreme drought in the EG site (*P* < 0.064). It is likely that the effect of oxygen on edaphic enzymes and *de novo* synthesis by the microbial community may have occurred within 5–24 hours of drought^18^. The lower sensitivity of extracellular enzymes compared with microbial metabolism to phenolics would disproportionately favor DOC release, rather than complete mineralization^18^. The increase of DOC in the EG site due to waterlogging may also be caused by the mobilization of released DOC from the peat matrix and persistence of β-glucosidase enzymes due to competitive inhibition. Indeed, there also appeared to be a declining gradient of DOC release by air drying due to drought legacy from the EG, DB2 and DB7 sites. Short-term flushing of DOC by drought and rewetting in the field has been observed in a number of studies^11, 12, 45, 50, 58^ and these results may explain some of the inconsistencies. Laine *et al*. observed higher DOC concentrations in drained than in pristine peat mesocosms in line with previous results that a deep aerobic layer promotes DOC release^59, 60^. Laine *et al*. also observed a lower pH and higher DOC in drained peat that was attributed to humic substances^61^ and carboxyl acids^62^ in accordance with our observations in the EG site at depth.

Phenolics consist of one aromatic ring and hydroxyl group and encompass low molecular compounds to complex highly polymerized compounds^63^. Phenolics exist as either (1) a dissolved form moving freely in the soil solution, (2) a sorbed form that reversibly binds to the soil particle or proteins, or (3) a polymerized form of phenolics consisting of humic substances each involving reversible sorption to soil through hydrophobic, hydrogen and ionic bonds^63^. Our study supports recent studies that suggest that the form of phenolics, not their chemical structure, can influence their fate in peat, as they cannot all be easily characterized into the slow recalcitrant pool in C dynamics^63^ or all be considered as potent inhibitors of hydrolase enzymes as according to the enzyme latch mechanism. Fierer found that low molecular phenolic compounds could serve as a labile substrate, promoting microbial biomass^64^. Müller *et al*. showed that lignin-drived phenolic compounds induced cellulase production, suggesting their potential to enhance decomposition^65^. Apparently the effect of phenolics on microbial extracellular enzyme activities depends on the source and composition rather than the absolute quantities^66^. In peatland ecosystems with a large amount of phenolics, higher phenol oxidase results in higher phenolic content in pore water as a product of enzyme action^63^. Bouskill *et al*. found evidence of an increase in the abundance of genes related to the oxidative enzymes targeting aromatic moieties in a forest soil drought experiment^19^. Wang *et al*. also observed a significant build-up of phenolics during short-term drought but suggested that reduced phenol oxidase activity as an explanation as supported by studies that show an optimal soil water content for phenol oxidase^10, 67^. Indeed, Wang *et al*. also found evidence of changes in phenolic quality and quantity in shrub/tree relative to *Sphagnum*/herb^10^. Therefore, long-term differences in vegetation succession from *Sphagnum* to shrub domination may also affect the characteristics of the peat and its decomposability by increasing inhibitory phenolics.

The observed reduction of *in vitro* β-glucosidase activity in surface peat by uncompetitive inhibition would have involved the inhibitor binding to the enzyme-substrate (ES) complex, resulting in the reduction in both *V*_*max*_ and *K*_*m*_. Kim *et al*. found that pine bark extract (containing phenols) caused a combination of non-competitive (decreased *V*_*max*_) and uncompetitive inhibition of α-glucosidase^68^. In the DB and WP sites at depth, inhibition was non-competitive, where the inhibitor reduces the activity (*V*_max_) of the enzyme and binds equally well to the enzyme whether or not it has already bound the substrate. Malcolm and Vaughan explained non-competitive inhibition of phosphomonoesterases by SOM in terms of conformational change in the enzyme structure when bound to humic material^69^. Our multivariate results suggest that uncompetitive (surface) and non-competitive (deep) inhibition were likely attributable to differences in H^+^ ions and SOM quantity and quality (phenolic content of humic substances) between depths. Humification was higher at depth suggesting a sustained microbial activity despite anoxic conditions resulting in higher concentrations of larger humic acids relative to smaller fulvic acids that represents the age of the deep peat layer. Indeed, the final stage of decomposition of humic matter is the breakdown of the more resistant lignoid or phenol part of the humic molecule and many carboxylic and phenolic acids are produced^35^. Wallage *et al*. also found that the surface peat layer consists predominately of fulvic acids (high E_4_/E_6_ ratio) from enhanced microbial decomposition and the lower depth of humic acids (lowE_4_/E_6_)^11^. Enzymes can be entrapped by humic molecules and can maintain their activity, being protected against proteolysis, thermal and pH denaturation, remaining active under conditions unfavorable for the activity of soil microorganisms^30, 34^.

Further research is required on enzyme kinetics using more realistic and dynamic laboratory experiments on moisture manipulations in a variety of peatlands exposed to varying hydrological legacy. The results presented here represent more extreme effects and there is a requirement to determine how enzyme inhibition may change in response to predicted climate change particularly moderate dry-rewetting cycles. In particular, analysis of the forms of phenolic compounds as well as *in vitro* enzyme kinetic and inhibition assays using specific fractionated or filtered humic/phenolic peat extracts. Phylogenetic and microbial functional diversity molecular approaches are required to establish how hydrological legacy impacts on the microbial community structure and function and *in situ* enzyme production using genomic, proteomic and metabolomic techniques to link these changes to extracellular enzyme kinetics.

In conclusion, enzyme kinetics and different types of enzyme inhibition were related to three-dimensional hydrological legacy suggesting that shifts in the microbial community structure and function over time alters the composition of humic compounds and their interaction with extracellular enzyme function. Importantly, the drought legacy site at depth following short-term drying and rewetting exhibited (1) a flush of DOC and phenolics that were (2) associated with increased β-glucosidase activity and (3) competitive inhibition. These results suggest that loss of carbon by short-term drought or rewetting may occur from sites with a legacy of drought due to the release of non-inhibitory phenolics that permit enhanced enzyme activity.

## Materials and Methods

### Site selection

The study was conducted at Geltsdale RSPB National Nature Reserve, North Pennines, UK (Fig. 1) because it is typical of British moorland or blanket bog and consists of areas of peatland with historically managed hydrological legacies or preserved in a semi-natural state by enclosures. The reserve (54°53′51″N, 2°34′28″W) is located at an altitude of 500 m and is dominated by ling heather (*Calluna vulgaris L*.), cotton grass (*Eriophorum* spp.), and bryophytes (*S. capillifolium*, *S. cuspidatum* and *S. papillosum*), and is described within the National Vegetation Classification^70^ as *C. vulgaris-Eriophorum vaginatum* blanket mire with *Empetrum nigrum* sub-community (M19b). Four sites were chosen with known historical hydrological legacies and flat slopes, all within the Geltsdale NNR (50 km^2^ catchment but sites were within 4 km^2^ of each other) as this reduced variation in climatic conditions (i.e. temperature, precipitation). Site 1 (eroded gully - EG) was located at Blackburn Head (54°54′11.46″N, 2°35′6.83″W) which was within a historically unmanaged area that had suffered from drainage due to gully erosion (i.e. unmanaged dry site). Site 2 (ditch-blocked <2 years - DB2) was located at Glendue Fell (54°53′15.91″N, 2°32′59.31″W) which had been drained historically but recently ditch-blocked within the last two years (i.e. short-term grip-blocked). Site 3 (ditch-blocked <7 years – DB7) was located at Halton Lea Fell (54°54′26.72″N, 2°32′52.14″W) which had also been drained historically but was known to have been ditch-blocked approximately seven years ago (i.e. long-term grip-blocked). Site 4 (wet pristine - WP) was located at Midgeholme Fell (54°54′27.23″N, 2°34′54.98″W) that had not been managed (except for burning in adjacent downslope locations) with potentially pristine mire hydrological conditions (i.e. unmanaged surface wet site with *Sphagnum* cover).

### Peat core collection and laboratory incubations

All peat cores were collected in spring 2010 between April and May. Samples were collected from the DB2 site at Coldfell on the 14/4/2010; at the DB7 site at Haltonlea Fell on the 21/4/2010; at the EG site at Blackburn Head on the 12/5/ 2010; and at the WP at Midgeholme Fell on the 18/5/2010. Four replicate sampling plots within each of the four sites were randomly selected for collecting peat cores, within areas that were visually representative of the site. At each replicate sampling plot, 3 peat cores were collected from the peat surface (0–10 cm) and 3 from depth (50–60 cm) – one for destructive baseline determinations, and two for incubating peat under either dry or waterlogged conditions in the laboratory. The 50–60 cm sampling depth was chosen to ensure samples were from the anaerobic, water saturated zone. A grab sample was also collected at each depth from the four plots for additional baseline determinations described below (see Supplementary Materials and Methods).

Peat cores and grab samples were returned to the laboratory on the same day as collection and stored at field temperature (8 °C) for 24 hours prior to analyses. Wet weights of all cores were determined. Eight cores were used for baseline determinations under the immediate site hydrological and thermal conditions for surface (n = 4) and deep (n = 4) peat including CO_2_ and CH_4_ production by headspace incubation with gas analysis by gas chromatography, bulk density and gravimetric water content (see Supplementary Materials and Methods). The 8 grab samples were used for determination of baseline pH, enzyme kinetics, and DOC as described in the sections below. The remaining 16 cores (8 from each depth) were all incubated at 8 °C in a temperature controlled incubator for 14 days in air (n = 4 per depth), or saturated with artificial rainwater in 1 litre Kilner jars (n = 4 per depth). Information on the artificial rainwater solution and storage of treatments is in Supplementary Materials and Methods.

After two weeks incubation, the dry cores were weighed. The water in the Kilner jars with the saturated cores was carefully removed along with the PVC tubes and the cores weighed. Bulk density determinations from the baseline replicates were used to estimate the gravimetric water content of the cores. CO_2_ and CH_4_ production were determined as described in Supplementary Materials and Methods. Peat pH, glucosidase and phenol oxidase enzyme kinetics, spectroscopy of water extracts and dissolved phenolics were determined as described in the sections below using a peat solution of 5 cm^3^ peat in 50 ml deionized water (see Supplementary Materials and Methods).

### Physicochemical conditions

Air and peat temperature were determined at each site on the day of collection using a hand-held meter with probe. Peat pH was determined using the peat solution with a standard pH electrode. The bulk density, gravimetric and volumetric water content of cores were determined by drying the cores of known volume in an oven at 105 °C for 24 hours.

### Organic matter pools

A known weight of dried peat was placed in a crucible and ignited at 550 °C for 24 hours to determine soil organic matter (SOM) content (% dry weight) by loss on ignition (LOI). 5 cm^3^ of peat was incubated in 50 ml of 0.1 M NaOH solution for 24 hours, filtered through 0.45 µm filter papers and stored at 4 °C before analysis. Samples were scanned between 400 and 700 nm using a scanning spectrophotometer. The E_4_/E_6_ ratio (humic/fulvic acids) was determined as the ratio of 465 to 665 nm^71^. The absorbance at 540 nm was also used as a measure of humification degree.

Dissolved organic carbon was extracted from the peat solution by centrifugation of subsamples at 10 000 rpm for 10 minutes, filtering through 0.45 µm filter papers and storing at 4 °C before analysis. Dissolved Organic Carbon (DOC) was determined on a TOC analyzer. Dissolved phenolic concentrations were determined using the Folin-Ciocalteau reagent microplate method^72^. Specific UV absorbance (SUVA_254_) was determined as the ratio of absorbance at 254 nm to DOC concentration and expressed as m^−1^ Abs_254_ mg^−1^ DOC L^−1^.

### Potential enzyme kinetics

The maximal rate of β-glucosidase enzyme reaction (*V*_max_) and Michaelis constant (*K*_m_) were determined using fluorogenic methylumbelliferyl (MUF)-β glucoside substrate according to the methods of Freeman *et al*.^73^ and Bonnett *et al*.^74^. Phenol oxidase activity (*V*_max_) was determined by the method of Pind *et al*.^75^ but not *K*_*m*_ as the rate of L-DOPA oxidation in soils may not vary linearly with time, substrate concentration or soil dilution^59^ (see Supplementary Methods and Materials for details of each assay).

### *In vitro* inhibition of pure β-glucosidase enzymes with peat

The inhibitive effect of peat from each site and hydrological treatment on kinetics of pure solutions of β-glucosidase were examined by mixing 1 U of pure β-glucosidase from almonds (Sigma-Aldrich G0395) in 1 ml^−1^ of peat extract in 1.5 ml microcentrifuge tubes. MUF β-glucopyranoside substrate was subsequently added to replicate reaction mixtures within 15 minutes between 0 and 500 µM. Enzyme kinetics were determined for each enzyme as described above with quench standards. Pure glucosidase in deionized water was used as a control to calculate % *V*_*max*_ and *K*_*m*_ of the control. Kinetic modelling procedures and methods for determining the types of enzyme inhibition are described below.

### Statistical Analysis

All statistical analyses were carried out using Minitab 13.20 (Mintab, Inc) or IBM SPSS Statistics Version 23.0.0.2. Data were tested for normality using the Kolmogorov-Smirnov normality test and homogeneity of variances using Bartlett’s test for normal data and Levene’s test for non-normal data. Data that failed the equal variance assumption were log transformed. The minimum value + 1 for negative datasets was added to each individual data point to shift the distribution from negative to positive values above 1 prior to transformation. Two-way General Linear Model ANOVA with Tukey multiple comparisons were used to compare Site x Depth factor interactions for each level of moisture under baseline conditions, and Site x Moisture factor interactions for each depth level (surface and deep). For enzyme kinetics, Michaelis-Menten coefficients (*V*_*max*_ and *K*_*m*_) and inhibition constants (*K*_*i*_) were determined using Michaelis-Menten and Substrate Inhibition non-linear regression models in GraphPad Prism 5. An F-test was used to accept the null hypothesis of Michaelis-Menten fit to the data or reject in favor of Substrate Inhibition. Identification of the inhibition mechanism for peat incubation with pure enzyme (uncompetitive, non-competitive, competitive or mixed) was achieved using plots of *K*_*i. NR*_ versus substrate [*S*] concentration according to Geng^76^. Based on the inhibition degree, [*R* = [*v*(+inhibitor)/*v*(−inhibitor)] = 1/(1 + [*I*]/K_i,NR_)], the apparent inhibition constant regardless of the inhibition mechanisms (K_i, NR_) could be calculated from *R* by rearranging this equation as K_i, NR_ = [*I*] *R*/(1 − *R*). Uncompetitive competition was identified by its unique decrease of *K*_*i. NR*_ with increase of [*S*] when [*S*] < *K*_*m*_. Competitive and non-competitive models were selected using Akaike’s Information Criterion (AIC). Prism calculates the difference in AICc values and the probability that each model is correct, with probabilities summing to 100%. Relationships between variables were tested using Spearman Rank correlation analysis and multiple stepwise regression analysis. Discriminant Function Analysis (DFA) was used to predict categorical groups of enzyme inhibition (competitive, uncompetitive and non-competitive) with continuous, independent decomposition variables. Principal Components Analysis (PCA) was used to determine relationships between decomposition pools and processes. All data from both depths and hydrological manipulations were included (*n* = 96).

## Electronic supplementary material

Supplementary Inofrmation
