# Supplementary material for: Hydrological legacy determines the type of enzyme inhibition in a peatlands chronosequence
Source: Sci Rep. 2017 Aug 30;7:9948. doi: 10.1038/s41598-017-10430-x (PMC5577268; doi:10.1038/s41598-017-10430-x)
Supplement: Supplementary file 1 — Supplementary Inofrmation [file 41598_2017_10430_MOESM1_ESM.doc]

**Hydrological legacy determines the type of enzyme inhibition**

**in a peatlands chronosequence**

Samuel Alexander Festing Bonnett, Edward Maltby and Chris Freeman

**Supplementary Materials and Methods**

*Peat core collection and laboratory incubations*

PVC tubes for core collection measuring 10 cm depth x 6 cm diameter were gently pushed into the peat following cutting with a knife, and carefully removed. Tubes and grab samples were stored in polyethylene bags for transport. In total, 24 peat cores and 8 grab samples were collected from each of the four sites. Samples were collected from the DB2 site at Coldfell on the 14/4/2010; at the DB7 site at Haltonlea Fell on the 21/4/2010; at the EG site at Blackburn Head on the 12/5/ 2010; and at the WP at Midgeholme Fell on the 18/5/2010.

The artificial rainwater consisted of the following chemical concentrations - 1.38 mg NH_4_NO_3_ l^-1^, 0.48 mg NH_4_Cl l^-1^, 0.4 mg KCl l^-1^, 3.26 mg MgSO_4_^.^7H_2_O l^-1^, 2.76 mg CaCl_2_.6H_2_O l^-1^, 4.12 mg NaCl l^-1^, and 0.64 mg Na_2_SO_4_ l^-1^. This solution was prepared to maintain natural acidity.

In the laboratory, the air dried cores were removed from their PVC tubes and supported on petri dishes using plastic mesh to maintain core structure. This reduced the water-holding capacity (WHC) to approximately 50 % over the incubation period. The water saturated cores remained in the PVC tubes to prevent erosion of peat core structure during the incubation period and maintained at 100 % WHC.

The peat solution was prepared by mixing 5 cm^3^ of peat from either a homogenized grab sample (baseline) or core (incubation) replicate in a 50 ml plastic centrifuge bottle to which deionized water was added up to the 50 ml mark. Samples were mixed with a spatula, shaken for 30 seconds, vortexed for a further 30 seconds, and filtered through a 2mm mesh.

*Physicochemical conditions*

Bulk density and SOM were not determined for hydrological manipulations as they were not expected to change significantly.

*CO_2_ and CH_4_ production*

In the laboratory, the cores were placed intact into 1 L Kilner glass jars to retain peat structure, giving a final headspace volume of 0.668 L, and were pre-incubated open in the dark for 24 hours for baseline measurements or 14 days for dry or wet (saturated) incubations at 8 °C to allow the conditions to stabilise. After the incubation period, the wet cores were drained of chamber water but remained fully water saturated, and the chambers were flushed with air and resealed. For all replicates, 30 ml of air was pumped into the headspace of each chamber with a syringe. The headspace was subsequently pumped five times and 30 ml of air was extracted and injected into a 25 ml pre-evacuated gas-tight vial and re-equilibrated to atmospheric pressure by venting briefly with a syringe needle. After one hour incubation, the headspace of each chamber was sampled as above. The one hour incubation period was chosen from preliminary timecourse analyses that showed linear gradients of CO_2_ and CH_4_ production from hourly measurements up to 2 hours. Gas samples were analysed for CO_2_ and CH_4_ using a Perkin Elmer Gas Chromatograph fitted with Plot Q columns connected to FID and ECD detectors in line with a backflush system to remove oxygen, and autosampler. The rate of production of each gas was determined by subtracting the headspace concentration at time zero from the final headspace concentration and dividing the difference by the dry weight and incubation period. Rates were expressed as µg g^-1^ h^-1^ for CH_4_ production, and mg CO_2_ g^-1^ h^-1^ for CO_2_ production.

*Potential enzyme kinetics*

For glucosidase kinetics, 1 ml of peat solution was placed in 10 x 2 ml micro-centrifuge vials. To each vial, deionized water and MUF substrate were added to give a range of substrate solutions between 0 (blank) and 200 µM for the surface samples and 0 – 1000 µM for the deep samples. Preliminary analysis had shown the surface enzyme curve to saturate at approximately 50 µM. To account for quenching, a reference MUF standard in water and a quench MUF standard in peat solution were prepared. Standards were prepared from a stock solution of 1000 µM MUF-free acid solution that was prepared by pre-dissolving in methylcellosolve using an ultrasonic bath. Buffer solution was not used in order to maintain field acidic conditions. Three batches of replicates were run together, shaken for 5 seconds and incubated at 8 ºC for 20 minutes that was within a pre-tested linear incubation phase. Samples were centrifuged at 12 000 rpm for 5 minutes and 300 µl of supernatant pipetted onto a 96 well plate for fluorescence determination at 365 nm excitation and 450 nm emission wavelengths using a BMG Labtech Galaxy fluorometer. Enzyme activities were calculated using the calculations presented in DeForest (2009) and expressed as MUF produced (nmol MUF g^-1^ peat dry weight h^-1^).

For phenol oxidase activity, 1 ml of peat solution was pipetted into 6 x 2 ml centrifuge vials. One ml of 10 mM L-DOPA solution was pipetted into 3 vials and the vials were shaken and incubated at 8 C for 5 minutes. One ml of deionized water was pipetted into the remaining vials and shaken. The reactions were terminated by centrifugation at 10000 rpm for 5 min and 300 µl of supernatant from each vial pipetted onto a 96 well plate and the absorbance was measured at 460 nm. The average of the three replicates for both water and substrate solutions were calculated and the activity determined from the difference. Activity was expressed in terms of nmol 2, 3-dihydroindole-5,6-quinone-2-carboxylate (diqc) per g dry weight per min, by using Beers Law a molar absorbancy of 3.7 x 10^4^.

**Supplementary Results**

*Site and peat physicochemical conditions*

**Table S1** Effect of drying and waterlogging on peat pH, water content, phenolic/DOC ratio, SUVA, E_4_/E_6_ ratio and humification rate (Abs. 540nm) in (a) surface and (b) deep peat. Significant differences between site baseline measurements (*P* < 0.05) are indicated by different numbers. Significant differences between baseline, dry and waterlogged conditions within each site are indicated by different letters. Mean ± standard error (*n* = 4).

a)

| **Site** | **Hydro-logical treatment** | **Gravimetric water content**  **(g g dry peat)** | **pH** | **Phenolics/DOC** | **SUVA_254_** | **E_4_/E_6_** | **Abs 540 nm** |
| --- | --- | --- | --- | --- | --- | --- | --- |
| EG | Baseline | 7.38 ± 0.741 ^1a^ | 3.8 ± 0.02 | 0.02 ± 0.011 ^a^ | 1.17 ± 0.147^1a^ | 6.16 ± 0.250 ^1^ | 0.18 ±0.030 |
|  | Dry | 4.14 ± 0.607 ^b^ | 3.8 ± 0.04 | 0.16 ± 0.011 ^b^ | 3.31 ± 0.266 ^b^ | 6.24 ± 0.117 | 0.14 ± 0.022 |
|  | Wet | 8.50 ± 0.989 ^a^ | 3.9 ± 0.03 | 0.15 ± 0.011 ^b^ | 3.34 ± 0.156 ^b^ | 6.16 ± 0.142 | 0.12 ± 0.018 |
| DB2 | Baseline | 7.84 ± 0.212 ^a^ | 3.9 ± 0.04 | 0.10 ± 0.015 | 0.75 ± 0.244 ^1^ | 6.52 ± 0.522 ^1^ | 0.19 ± 0.040 |
|  | Dry | 3.43 ± 0.153 ^b^ | 3.9 ± 0.02 | 0.06 ± 0.026 | 0.92 ± 0.402 | 6.30 ± 0.262 | 0.28 ± 0.009 |
|  | Wet | 8.75 ± 0.349 ^a^ | 4.0 ± 0.05 | 0.05 ± 0.026 | 0.56 ± 0.126 | 6.04 ± 0.255 | 0.15 ± 0.008 |
| DB7 | Baseline | 10.48 ± 0.758 ^2a^ | 4.0 ± 0.03 ^1^ | 0.07 ± 0.042 | 0.84 ± 0.337 ^1^ | 4.21 ± 0.348 ^2a^ | 0.16 ± 0.018 |
|  | Dry | 4.660 ± 0.169 ^b^ | 3.8 ± 0.07 | 0.05 ± 0.011 | 1.26 ± 0.232 | 6.83 ± 0.430 ^b^ | 0.12 ± 0.017 |
|  | Wet | 11.90 ± 0.901 ^a^ | 3.9 ± 0.04 | 0.05 ± 0.022 | 0.78 ± 0.166 | 6.90 ± 0.460 ^b^ | 0.07 ± 0.013 |
| WP | Baseline | 10.54 ± 0.409 ^2a^ | 3.7 ± 0.08 ^2^ | 0.10 ± 0.008 | 2.74 ± 0.179 ^2^ | 6.49 ± 0.263 ^1^ | 0.14 ± 0.016 ^a^ |
|  | Dry | 3.320 ± 0.776 ^b^ | 3.8 ± 0.04 | 0.15 ± 0.044 | 2.42 ± 0.202 | 6.16 ± 0.141 | 0.41 ± 0.079 ^b^ |
|  | Wet | 12.71 ± 0.822 ^a^ | 3.9 ± 0.04 | 0.18 ± 0.020 | 2.56 ± 0.098 | 6.15 ± 0.233 | 0.42 ± 0.106 ^b^ |

b)

| **Site** | **Hydro-logical treatment** | **Gravimetric water content**  **(g g dry peat)** | **pH** | **Phenolics/DOC** | **SUVA_254_** | **E_4_/E_6_** | **Abs 540 nm** |
| --- | --- | --- | --- | --- | --- | --- | --- |
| EG | Baseline | 10.92 ± 0.280 ^a^ | 3.8 ± 0.02 ^a^ | 0.02 ± 0.010^1a^ | 0.58 ± 0.108^1a^ | 3.78 ± 0.122 | 0.31 ± 0.021 |
|  | Dry | 5.98 ± 0.496 ^b^ | 3.5 ± 0.02 ^b^ | 0.26 ± 0.012^b^ | 3.35 ± 0.069^b^ | 3.73 ± 0.061 | 0.36 ± 0.016 |
|  | Wet | 11.77 ± 0.324 ^a^ | 3.8 ± 0.03 ^a^ | 0.34 ± 0.051^b^ | 4.62 ± 0.347^b^ | 3.86 ± 0.061 | 0.24 ± 0.014 |
| DB2 | Baseline | 7.88 ± 0.632 ^a^ | 3.9 ± 0.04 | 0.09 ± 0.014 | 0.60 ± 0.127^1^ | 4.72 ± 0.108 | 0.22 ± 0.022 |
|  | Dry | 4.29 ± 0.636 ^b^ | 3.7 ± 0.07 | 0.09 ± 0.018 | 0.51 ± 0.072 | 4.16 ± 0.166 | 0.55 ± 0.047 |
|  | Wet | 8.41 ± 1.100 ^a^ | 4.0 ± 0.03 | 0.07 ± 0.014 | 0.50 ± 0.195 | 4.09 ± 0.144 | 0.26 ± 0.025 |
| DB7 | Baseline | 9.87 ± 0.828 ^a^ | 4.0 ± 0.03 | 0.05 ± 0.017 | 0.41 ± 0.099^1^ | 3.84 ± 0.172 ^a^ | 0.29 ± 0.012 |
|  | Dry | 5.89 ± 0.576 ^b^ | 3.7 ± 0.06 | 0.07 ± 0.010 | 1.58 ± 0.250 | 5.14 ± 0.477 ^b^ | 0.20 ± 0.067 |
|  | Wet | 11.14 ± 1.060 ^a^ | 3.9 ± 0.02 | 0.09 ± 0.049 | 0.71 ± 0.288 | 5.08 ± 0.383 ^b^ | 0.13 ± 0.019 |
| WP | Baseline | 8.72 ± 0.584 ^a^ | 3.9 ± 0.01 | 0.18 ± 0.039^2^ | 3.12 ± 0.077^2^ | 4.42 ± 0.078 | 0.29 ± 0.019 |
|  | Dry | 2.98 ± 0.727 ^b^ | 3.7 ± 0.02 | 0.23 ± 0.033 | 3.80 ± 0.698 | 4.73 ± 0.215 | 0.20 ± 0.067 |
|  | Wet | 9.88 ± 1.364 ^a^ | 3.9 ± 0.03 | 0.27 ± 0.034 | 4.18 ± 0.361 | 4.56 ± 0.041 | 0.18 ± 0.043 |

**Table S2** Non-linear regression model outputs for uncompetitive, competitive and noncompetitive inhibition. Models were selected based on AIC probability and *R^2^*. Note that different inhibition constants (mean ± standard error) are given for uncompetitive (*ak* *_i_*) and competitive and noncompetitive inhibition (*k_i_*).

| **Site** | **Depth** | **Moisture** | **Inhibition constants** | **Inhibition model** | **AIC Probability (%)** | ***R^2^*** | **Alternative** | ***R^2^*** |
| --- | --- | --- | --- | --- | --- | --- | --- | --- |
| EG | Surface | Dry | *ak_i_* = 0.093 ± 0.0078 | Uncompetitive |  | 0.65 |  |  |
|  |  | Wet | *ak_i_* = 0.092 ± 0.0074 | Uncompetitive |  | 0.78 |  |  |
|  | Deep | Dry | *k_i_* = 0.016 ± 0.0013 | Competitive | 99.99 | 0.94 | Noncompetitive | 0.65 |
|  |  | Wet | *k_i_* = 0.011 ± 0.0011 | Competitive | 99.64 | 0.90 | Noncompetitive | 0.72 |
| DB2 | Surface | Dry | *ak_i_* = 0.115 ± 0.0091 | Uncompetitive |  | 0.80 |  |  |
|  |  | Wet | *ak_i_* = 0.119 ± 0.0094 | Uncompetitive |  | 0.75 |  |  |
|  | Deep | Dry | *k_i_* = 0.117 ± 0.0121 | Noncompetitive | 99.74 | 0.57 | Competitive | 0.29 |
|  |  | Wet | *k_i_* = 0.175 ± 0.0122 | Noncompetitive | 99.99 | 0.87 | Competitive | 0.53 |
| DB7 | Surface | Dry | *ak_i_* = 0.072 ± 0.0071 | Uncompetitive |  | 0.75 |  |  |
|  |  | Wet | *ak_i_* = 0.067 ± 0.0069 | Uncompetitive |  | 0.83 |  |  |
|  | Deep | Dry | *k_i_* = 0.076 ± 0.0087 | Noncompetitive | 94.73 | 0.79 | Competitive | 0.57 |
|  |  | Wet | *k_i_* = 0.076 ± 0.0090 | Noncompetitive | 93.00 | 0.88 | Competitive | 0.64 |
| WP | Surface | Dry | *ak_i_* = 0.041 ± 0.0078 | Uncompetitive |  | 0.56 |  |  |
|  |  | Wet | *ak_i_* = 0.050 ± 0.0078 | Uncompetitive |  | 0.64 |  |  |
|  | Deep | Dry | *k_i_* = 0.031 ± 0.0093 | Noncompetitive | 50.94 | 0.65 | Competitive | 0.63 |
|  |  | Wet | *k_i_* = 0.048 ± 0.0094 | Noncompetitive | 86.43 | 0.93 | Competitive | 0.39 |


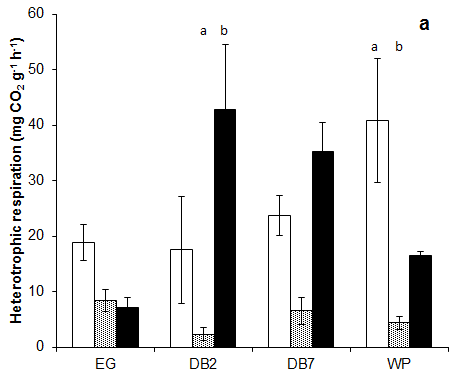


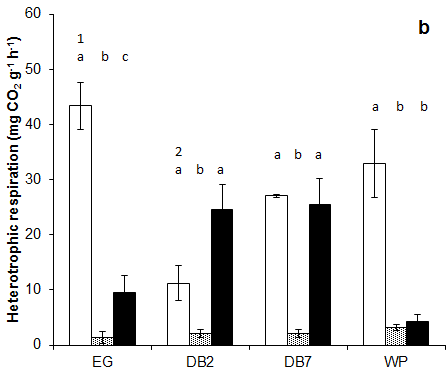


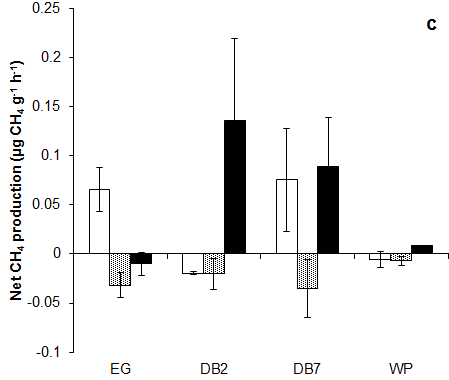


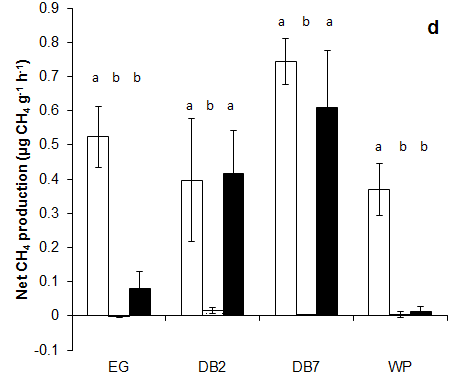


**Figure S1 Heterotrophic respiration within (a) surface peat and (b) peat at depth and net CH_4_ production within (c) surface peat and (d) peat at depth.** White bars = baseline measurements, grey bars = air dried treatment and black bars = waterlogged treatment. Significant differences between site baseline measurements (P < 0.05) are indicated by different numbers. Significant differences between baseline, dry and waterlogged conditions within each site are indicated by different letters. Mean ± standard error (n = 4).


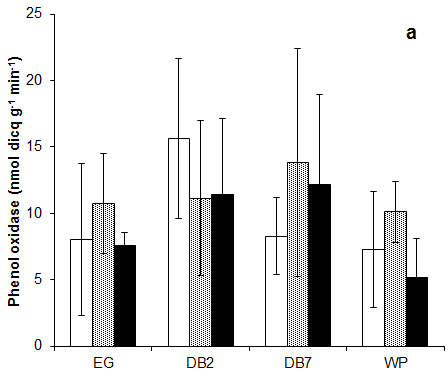


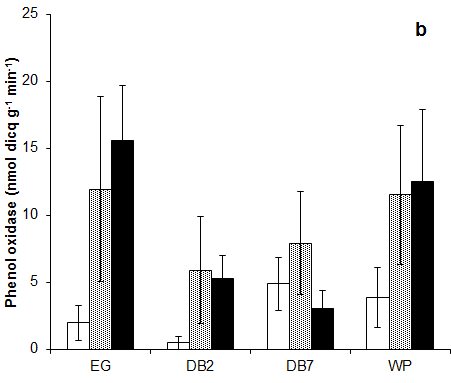


**Figure S2 Phenol oxidase activity within (a) surface peat and (b) peat at depth.** White bars = baseline measurements, grey bars = air dried treatment and black bars = waterlogged treatment.There were no significant differences. Mean ± standard error (n = 4).


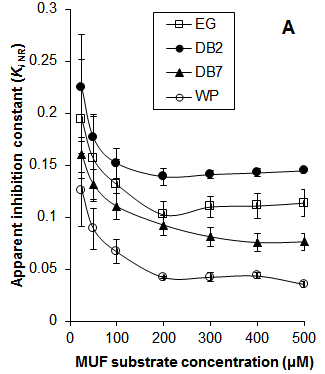

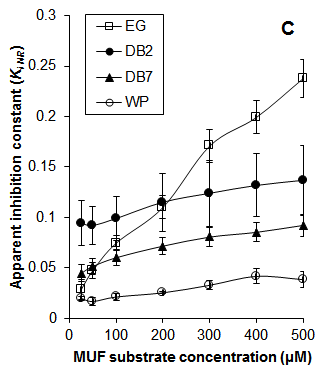


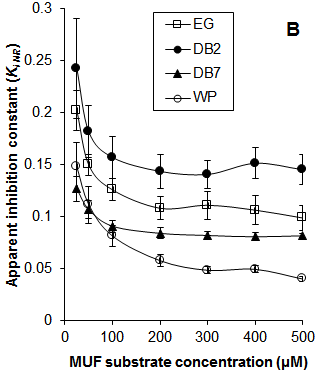

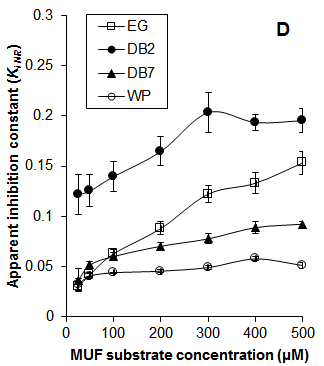


**Figure S3** Apparent inhibition constants (*K_i NR_*) for each site showing uncompetitive (decrease of *K_i NR_* values with the increase of [*S*] when [*S*] < *K*_m_) versus competitive and non-competitive inhibition (positive linear) relative to MUF substrate concentration under (a) surface + dry, (b) surface + wet, (c) deep + dry and (d) deep + wet conditions.

**Table S3** Standardized Canonical Discriminant Function Coefficients, Eigenvalues and Wilks’ Lambda for groups according to type of enzyme inhibition. Of the original grouped cases, 92.7 % were correctly classified (Group 1: Uncompetitive inhibition = 95.8%; Group 2: Mixed inhibition = 94.4%; Group 3: Competitive inhibition = 75.0%).

|  | **Function 1** | **Function 2** |
| --- | --- | --- |
| [H^+^] | -0.051 | 0.999 |
| Moisture | 0.194 | 0.638 |
| SOM | -0.731 | -0.485 |
| NaOH E_4_E_6_ | 0.699 | -0.121 |
| NaOH Abs. 254 | 0.105 | 0.279 |
| DOC | 0.067 | -1.063 |
| Phenolics | -0.307 | 0.964 |
| SUVA | 0.189 | 0.091 |
| Eigenvalue | 4.042 | 0.641 |
| % of variance | 86.30 | 13.70 |
| Canonical Correlation^2^ | 0.801 | 0.391 |
| Wilks’ Lambda | 0.121 | 0.609 |
| Significance | 0.001 | 0.001 |

**Supplementary References**

DeForest JL. (2009) The influence of time, storage temperature, and substrate age on potential soil enzyme activity in acidic forest soils using MUB-linked substrates and L-DOPA. Soil Biol Biochem 41: 1180-1186.
